# Intra-iliac bone marrow injection as a novel alternative to intra-tibial inoculation in rat model

**DOI:** 10.1186/s13287-021-02413-7

**Published:** 2021-06-10

**Authors:** Marwa S. Khattab, Huda O. AbuBakr, Kassem G. El Iraqi, Naglaa A. AbdElKader, Mervat M. Kamel, Khaled Hamed Salem, Julia Steitz, Mamdouh Afify

**Affiliations:** 1 Department of Pathology, Faculty of Veterinary Medicine, Cairo University, Giza, 12211 Egypt; 2 Department of Biochemistry and Molecular Biology, Cairo University, Giza, Egypt; 3 Department of Veterinary Hygiene and Management, Cairo University, Giza, Egypt; 4 Department of Surgery, Anesthesiology & Radiology, Faculty of Veterinary Medicine, Cairo University, Giza, Egypt; 5 Department of Orthopedic Surgery, Faculty of Medicine, Cairo University, Giza, Egypt; 6 Department of Orthopedic Surgery, RWTH Aachen University Faculty of Medicine, Aachen, Germany; 7 Institute for Laboratory Animal Science, RWTH Aachen University Faculty of Medicine, Aachen, Germany

**Keywords:** Intra-bone marrow injection, Intra-iliac injection, Animal welfare, Stress hormones, Radiology, Behavior, Histopathology

## Abstract

**Background:**

Intra-bone marrow injection (IBMI) in rats is adopted in many studies for stem cell and hematopoietic cell transplantation. IBMI in the tibia or the femur results in severe distress to the animal. Therefore, this study aims to evaluate intra-iliac injections as an alternative approach for IBMI.

**Methods:**

Twenty-seven Sprague Dawley rats were assigned into 3 groups, 9 rats each, for 4 weeks. The control group rats were not injected. Tibia group rats were injected intra-tibial and the iliac group rats were injected intra-iliac with saline. Behavioral, radiological, histopathological, and stress evaluation was performed. Total bilirubin, cortisol, and insulin-like growth factor-1 (IGF1) were measured.

**Results:**

Behavioral measurements revealed deviation compared to control, in both injected groups, on the 1st and 2nd week. By the 3rd week, it was equivalent to control in the iliac group only. Bilirubin and cortisol levels were increased by intra-tibial injection compared to intra-iliac injection. The IGF-1 gene expression increased compared to control at 1st and 2nd weeks in intra-iliac injection and decreased by intra-tibial injection at 2nd week. The thickness of the iliac crest was not different from the control group, whereas there were significant differences between the control and tibia groups. Healing of the iliac crest was faster compared to the tibia. In the 3rd week, the tibia showed fibrosis at the site of injection whereas the iliac crest showed complete bone reconstruction.

**Conclusion:**

Intra-iliac injections exert less distress on animals, and by 3 weeks, they regained their normal activity in comparison to intra-tibial injections.

## Background

Intra-bone marrow injection (IBMI) has been adopted in many studies involving stem cells and hematopoietic cell transplantation [1–4]. A previous trial showed that IBMI can reduce non-specific cell loss and allow better seeding efficiency of transplanted cells than intravenous injection [4]. Intra-bone marrow injection was usually performed in the tibial shaft of mice and rarely in the femur due to its easier technique [5]. However, the IBMI in the tibia causes severe post-injection distress, and a higher degree of impairment compared to IBMI in the femur [6]. Moreover, bone marrow aspiration in experimental rats was performed from the tibia or femur. To harvest enough quantities of bone marrow, the researchers usually choose to kill the animal to obtain bone marrow from the femur or tibia. Several previous studies described techniques that can allow bone marrow aspiration from the iliac crest and sparing the animal life [7, 8]. Furthermore, many studies used injection in mice and rats in tumor models [9, 10], which were associated with severe distress to the animals. Also, the follow-up of the disease process, especially at an earlier stage, induces additional stress to animals, without precious results [11].

The pain and distress caused by various techniques of IBMI can be assessed by biomarkers such as total bilirubin, cortisol, and insulin-like growth factor (IGF-1), which is one of the fundamental factors in skeletal growth during puberty and bone health throughout life. The latter plays a central role in cellular growth, differentiation, survival, and cell cycle progression, which allows its use in the assessment of the beneficial effect of injection techniques without distress at its site [12–14].

The degree of distress of IBMI in the tibia and iliac crest of rat and their impact on animal welfare has not yet been evaluated. Therefore, this study will assess the impact of IBMI on the tibia and iliac crest in this regard, without their exposure to excessive manipulation, based on our experimental findings.

## Materials and methods

### Animals and housing

#### Rats

Twenty-seven male Sprague Dawley rats were obtained and reared in the Laboratory Animals Unit of Animal Behavior and Management, Department of Veterinary Hygiene and Management at Faculty of Veterinary Medicine, Cairo University, Egypt. The rats weighing average was 120 g, housed in plastic shoebox-type cages (37×27×17 cm) with a floor space of approximately 1000 cm^2^/ cage, stainless steel wire lids, and sawdust as a bedding material. The rats were kept at a temperature of 30–35.5°C and relative humidity of 55–65%. Lighting was maintained on a natural daylight regime and no artificial light was used except at the time of observation.

#### Management practices

Individual identification by using the color marking technique with a dye that is applied to the tail region. A commercial balanced diet was offered to rats. Feed was offered to animals ad libitum twice a day (8 am and 2 pm). A continuous adequate supply of clean fresh water was available all day.

### Experimental work

The animals were allocated into nine cages (three rats/cage). The rats were classified into three groups: un-injected control group, tibial group injected intra-tibial and iliac group injected intra-iliac with saline. The experiment was conducted for 4 weeks. The weight of animals was taken at the beginning of the experiment (initial weight).

### Surgical approach

**Fig. 1:**
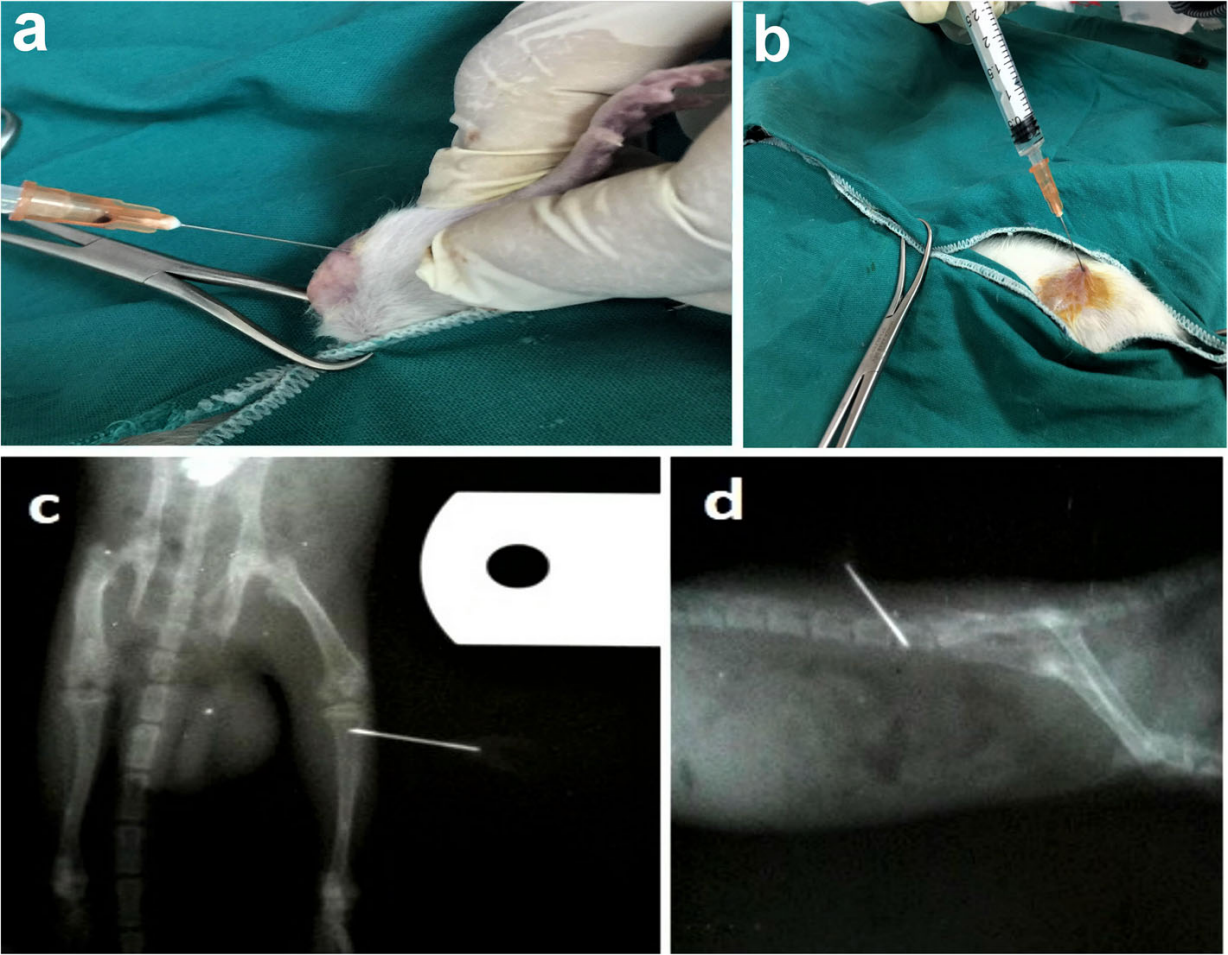
Macroscopic and radiologic approach of the route of intraosseous injection. **a**, **c** Intra-tibial injection. **b**, **d** Intra-iliac injection

The animals were weighed and anesthetized intraperitoneally with a combination of 2% Xylazine (Rompun; Bayer, Germany; 10 mg/kg body weight) and 10% Ketamine (Germany; 100 mg/kg body weight) [15, 16]. The Xylazine and Ketamine were diluted before administration with 0.9% saline. Ketamine Xylazine mixture caused a profound analgesic effect and relieved the post-injection pain [17]. The volume administrated was based on the dosage and weight of the animal. After shaving and applying aseptic precautions to the proximal part of the tibia (tibial tuberosity) of nine rats, a sterile needle of 5-mL syringe (22 gauge) was inserted for puncturing the skin and drilling into the tibial tuberosity, followed by injecting 0.3-mL sterile ringer’s solution using a 3-mL syringe (25 gauge) (Fig. 1a). After shaving and taking aseptic precautions to the lateral aspect of ilium of nine rats to inject in the iliac crest, it was punctured by a sterile needle of 3-mL (25 gauge) syringe after the full extension of hind paws backward to clarify the point of injection. It was followed by injecting a 0.3-mL sterile ringer’s solution (Fig. 1b). To verify correct needle positioning, an x-ray was performed (Fig. 1c and d).

### Body weight (BW) and body weight gain

Rats were weighed on the day of arrival and once every week, at a fixed time, along the course of the experiment. Bodyweight gain was calculated by determining the difference between the average body weight of rats measured in this particular week (W2) and the average body weight of rats measured in the previous week (W1) by the following equation. Bodyweight gain = (W_2_ − W_1_).

### Behavioral measurement

The experimental rats were trained for the behavior tests as open field, beam walking, beam balance, and footprint test over 5 days before the surgical operation and continued 3 weeks post-injection for 3 days a week (every second day). The behavioral tests were done during the light phase; all devices were cleaned in-between with a 75% ethanol solution and permitted to be dry.

#### Open field (OF) test

The open field test was conducted to assess the emotional behavior of the rat with different routes of injection. The open field apparatus was in the form of a square wooden arena measured (90 × 90 × 25cm). The wood of the apparatus was covered by a plastic laminate to prevent fluids (e.g., urine) absorption. The floor was divided by black lines into 36 small squares (15×15cm). Rats were gently placed into a corner of the arena and kept for 3 min [18]. During the test duration, anxiety was assessed by measuring immobility (Time spent freezing) and vertical activity (exploratory behaviors in the form of ambulation and crossing of squares and rearing). In addition, non-exploratory measures comprising grooming, and eliminative behaviors (number of fecal balls “defecation” and the number of urination spots), were evaluated [19].

#### Beam walking test

A relatively new hind limb test that was developed is the ledged tapered beam-walking. It examines the ability of a rat to cross a narrow, elevated beam of wood of 105 cm long, 4 cm wide, and 3 cm high [20]. The beam was suspended 80 cm from the ground by wooden supports. The wooden supports at the “starting” end of the beam formed a sheer drop while a platform was located at the other end to which the black home cage was placed. The time was recorded when the rat placed a weight-bearing step entirely over the start line and represented the latency to begin the task. The stopwatch was then stopped when all four feet were placed entirely upon the finishing platform at the opposite end of the beam. The maximum time allowed for the task was 2 min [21].

#### Beam balance test

Rats were placed at the center of a 1.5-cm-wide square wooden bridge that was suspended 60 cm above the ground. All animal groups were tested (one trial/rat) for 3 days per week within 3 weeks scored from 1 to 4 on beam balance [22]. All trial scores from each group were averaged weekly for statistical analysis. The scoring was as follows: 1, balances with freezing; 2, balances and walking; 3, hugs the beam or slips without falling; and 4, drops over the beam or hangs on the beam and falls off. The results were calculated as a percentage for each group.

#### Footprint

Rat gait analysis was done by collecting images of its footprints, after smearing the toes of forelimbs with red paint and the toes of hind limbs with blue paint, then allowed to walk down the track, leaving its footprints on normal white paper [23]. Stride length and width from the paper were measured all over the experimental period.

### Samples

**Fig. 2:**
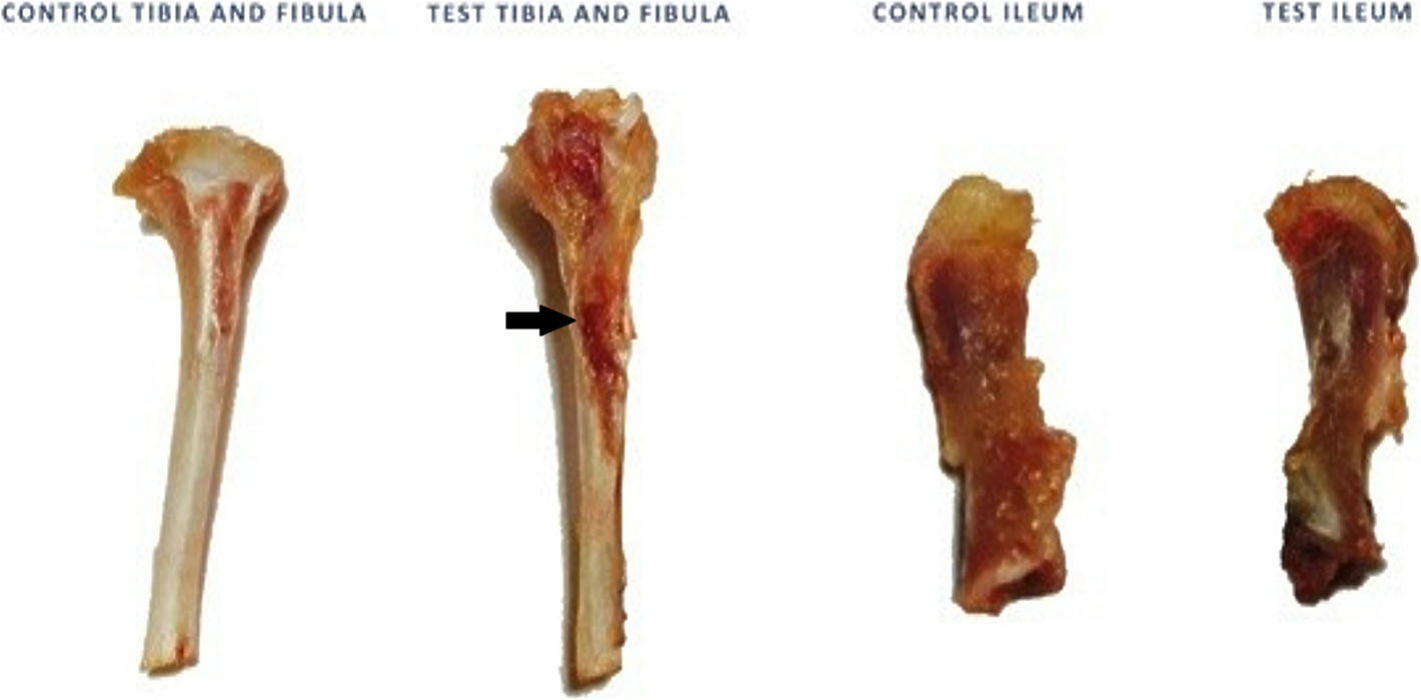
Bone samples collected at autopsy at 1 week showing the difference between control tibia and test tibia. The tibial tuberosity is congested in the test tibia (black arrow), whereas the ilium has a nearly similar iliac crest in control and test ilium with only mild reaction in test ileum.)

At the end of each week, blood samples were collected from the medial canthus of the eye after the animal was intraperitoneally injected with a combination of 2% Xylazine (Rompun; Bayer, Germany; 10 mg/kg body weight) and 10% Ketamine (Germany; 100 mg/kg body weight). A portion of samples was collected on EDTA-containing tubes and the other portion was left to clot in clear dry centrifuge tubes, then centrifuged at 3500 r.p.m for 15 min for serum preparation. Both serum and whole blood samples were stored at −80 °C, for biochemical and gene expression analysis respectively. Bone samples from the iliac crest and tibia were grossly examined and then fixed in 10% neutral buffered formalin (Fig. 2).

### Biochemical analysis

Serum total bilirubin was measured according to the manufacturer’s protocols using a total bilirubin kit (Sigma Aldrich) to evaluate the stress of the different types of injections. A serum stress hormone, cortisol, was determined by using an enzyme-linked fluorescent assay according to the manufacturer’s protocol (VIDAS® Cortisol S; BIOMERIEUX company).

### Quantitative real-time PCR evaluation for insulin-like growth factor 1 (IGF-1) expression

Blood total RNA isolation was performed using a QIAmp RNA mini kit (Qiagen, Hilden, Germany) according to the manufacturer’s manual. The concentration and purity of the total RNA samples were obtained by using a Nanodrop ND-1000 spectrophotometer. The isolated RNA was used for cDNA synthesis using reverse transcriptase (Fermentas, EU). Real-time PCR (qPCR) was carried out using the reaction mixture of 1 μL cDNA, 0.5mM of each primer (IGF-1; forward primer: TCTCCTAGTCCCTGCCTCTT, reverse primer: TCTGTGAAGGAAGCGGCTTA), (GAPDH; forward primer: GAGACAGCCGCATCTTCTTG, reverse primer: TGACTGTGCCGTTGAACTTG 3′), Maxima SYBR Green/Rox qPCR Master Mix (Thermo Scientific) in a total volume of 20 μL. PCR amplification and analysis were achieved using Bio-Rad Cycler thermal cycler and the MyiQ real-time PCR detection system. Each assay includes triplicate samples for each tested cDNAs and no-template negative control. The ∆CT value was calculated by the subtraction of the GAPDH CT, as an internal control from each gene CT, in which CT is the cycle number at which detectable signals are achieved [24].

### Histopathology and immunohistochemistry

Fixed bone was decalcified in a 10% HCL and 10% formic acid solution [25] for 7 days and the decalcified samples were processed using paraffin embedding technique. Tissue sections of 5 μm were made using a microtome (Leica 2135) and were stained by hematoxylin and eosin stain (HE) [26]. Stained tissue sections were examined by a light microscope and photographed using an Olympus camera (Tokyo, Japan). A modified semi-quantitative scoring system was performed in which hemorrhage, inflammatory cells, necrosis, disrupted trabeculae, fibrosis, and capillary proliferation were assessed on a scale from 1 to 5 [27] (1, non/negligible; 2, mild; 3, moderate; 4, moderate to severe; 5, severe). Immunohistochemistry for macrophages (CD68, a primary monoclonal antibody concentration of 1:400) and proliferation marker (Ki67, primary monoclonal antibody concentration of 1:100) was carried out on paraffin tissue sections (Immunhisto. Dako LSAB Kit from Agilent, CA 95051, USA).

### Statistical analysis

Statistical analysis was done using the Statistical Package for Social Science (SPSS Win version 17, 2007) and the interpretation of the data was done according to Patrie and Watson [28].

Parametrical statistical tests were applied (unpaired Student’s T-test, one-way and two-way analysis of variance (ANOVA) after the exploration of the data. Descriptive statistics are represented by means ± standard deviation (SD) and percentages. The least significant difference (LSD) was used for significance among groups at the significance level (*p*<0.05). Lesion scores were assessed by non-parametric test (Kruskal-Wallis test) to detect significance at *p*<0.05 and significance between groups was detected by Mann-Whitney test.

## Results

### Behavioral findings

#### Recovery from anesthesia

The appropriate anesthetic management is essential for achieving success in the surgical operation. Induction and recovery time were evaluated in each animal. The time of anesthesia and recovery was not significantly different between groups; however, the operation time was longer in the ilium group (Table 1).

**Table 1:** Time taken in injection and recovery from anesthesia

Time in minutes	Parameter	
	Intra-iliac injection	Intra-tibial injection
Time of anesthesia	2.77 ± 0.43^a^	2.44 ± 0.17^a^
Operation time	3.33 ± 0.44^a^	2.22 ± 0.27 ^b^
Recovery time	93.33 ± 11.31^a^	71.88 ± 9.92 ^a^

^a^ and ^b^ letters within the raw mean significant difference at p≤ 0.05 between the groups. The results are expressed as mean ± Standard Error

#### Bodyweight gain (physical performance)

There was a significantly low body weight gain in IBMI rats (intra-tibial and intra-iliac) in the first week post-surgery, compared to the control group, while in the second and third weeks, there was a significant (*p*≤ 0.05) improvement in body weight gain in rats injected intra-iliac than those injected intra-tibial (Table 2).

**Table 2:** Performance parameters within weeks for different groups

Time	Parameter	Injection group			*P*-value
		Control	Intra-tibial	Intra-iliac	
First week	Initial weight	120.55 ± 5.85^a^	120.77± 5.78^a^	127.77 ± 7.86^a^	0.24
	Final weight	144.44 ± 7.74^a^	137.55 ± 6.20^b^	134.44± 10.25^b^	0.05
	Body weight gain	23.88 ± 9.04 ^a^	16.88± 3.34^b^	6.66 ± 11.75^c^	0.05
Second week	Initial weight	144.44 ± 7.74^a^	137.55 ± 6.20^b^	134.44± 10.25^b^	0.05
	Final weight	170±0.83^a^	158.33± 7.49^b^	176.66±10.92^a^	0.03
	Body weight gain	25.56± 8.70^b^	20.78± 13.29^b^	42.22± 13.64^a^	0.001
Third week	Initial weight	170±0.83^a^	158.33± 7.49^b^	176.66±10.92^a^	0.03
	Final weight	190 ± 8.66^a^	168.33± 4.40^b^	198.33 ± 6.66^a^	0.002
	Body weight gain	20.0 ± 5.77^a^	10.0 ± 13.22^b^	21.67± 18.33^a^	0.01

Result expressed as mean ± standard error. Values bearing different superscripts indicate significance between the groups. The least significant difference (LSD) was used for significance among groups at the significance level (*p *< 0.05)

#### Open field test

Locomotor activity in the open field arena of rats via the intraosseous route is recorded in Table 3. In the first week, both injected groups showed a significant (*p*≤ 0.05) decrease in distance traveled/ horizontal activity (number of squares crossed) and vertical activity (rearing) in the open field when compared with un-injected rats. However, in the second week, all these measures were increased significantly (*p*≤ 0.05) in all rats injected intra-iliac when compared to rats belonging to the intra-tibial group. In the third week, no differences were recorded between the injected and control groups.

**Table 3:** The open field test within weeks for different groups

Time	Parameter	Injection group			*P*-value
		Control	Intra-tibial	Intra-iliac	
First week	Freezing/sec.	6.00 ± 1.1^b^	28.55± 12.14^ab^	41.66 ± 13.7^a^	0.037
	Peripheral activity	19.66 ± 4.11^a^	16.29 ± 4.37 ^a^	12.22 ± 2.04^a^	0.36
	Central activity	1.14 ± 0.41^a^	0.22 ± 0.15^b^	0.0 ± 0.0^b^	0.01
	Rearing	2.44 ± 0.61^a^	1.63 ± 0.46^a^	1.62 ± 0.34^a^	0.25
	Grooming	2.22 ±0.56^a^	2.03 ± 0.46^a^	1.55 ± 0.31^a^	0.37
	Defecation	0.92 ± 0.18^a^	1.4 ± 0.37^a^	1.7 ± 0.57^a^	0.47
	Urination/Nu.	0.33 ± 0.07^a^	0.25 ± 0.07^a^	0.46 ± 0.14^a^	0.6
Second week	Freezing/sec.	19.0± 14.43^a^	14.58 ± 3.35^b^	18.08 ± 9.64^a^	0.05
	Peripheral activity	14.41± 2.43^b^	13.5 ± 2.97^b^	23.08 ± 4.77^a^	0.01
	Central activity	0.08 ± 0.08^a^	0.0 ± 0.0^a^	0.0 ± 0.0^a^	0.48
	Rearing	0.91 ± 0.39 ^b^	1.16 ± 0.35^b^	3.5 ± 1.32^a^	0.043
	Grooming	0.83 ± 0.16^a^	1.17 ± 0.21^a^	1.0 ± 0.34^a^	0.53
	Defecation	2.08 ± 0.35^a^	3.08 ± 0.57^a^	1.83 ± 0.53^a^	0.16
	Urination/Nu.	0.0 ± 0.0^a^	0.08 ± 0.08^a^	0.08 ± 0.08^a^	0.64
Third week	Freezing/sec.	2.66 ± 0.33^c^	7.33 ± 4.58^a^	4.0 ± 0.86^b^	0.04
	Peripheral activity	12.66 ± 2.9^a^	8.66 ± 2.12^b^	9.67 ± 2.02^b^	0.02
	Central activity	0.66 ± 0.6^a^	0.0 ± 0.0^a^	0.0 ± 0.0^a^	0.44
	Rearing	0.0 ± 0.0^a^	0.17 ± 0.17^a^	0.0 ± 0.0^a^	0.44
	Grooming	2.00 ± 0.57^a^	0.0 ± 0.0^b^	0.0 ± 0.0^b^	0.02
	Defecation	2.66 ± 1.76^a^	2.83 ± 1.58^a^	2.67 ± 0.72^a^	0.99
	Urination/Nu.	0.0 ± 0.0^a^	0.5 ± 0.28^a^	0.16 ± 0.016^a^	0.28

Result expressed as mean ± standard error. Values bearing different superscripts indicate significance between the groups. The least significant difference (LSD) was used for significance among groups at the significance level (*p *< 0.05)

Also, the anxiety state of the rats was assessed on the first occurrence in the open field arena as shown in Table 3. Anxiogenic effects were measured in rats injected intra-osseous and un-injected control rats in the open field test throughout the 3 weeks of study. Rats exposed to injection either in the tibia or in the ilium showed a marked significant (*p*≤ 0.05) increase in freezing time and fecal boli when compared to rats belonging to the control group in the first week post-injection. The time spent freezing and defecation scores were decreased in rats injected intra-iliac in the second week than others in the tibial group. On the other hand, our treatments had no significant influence on urination scores during the study.

#### Beam walking test

**Fig. 3:**
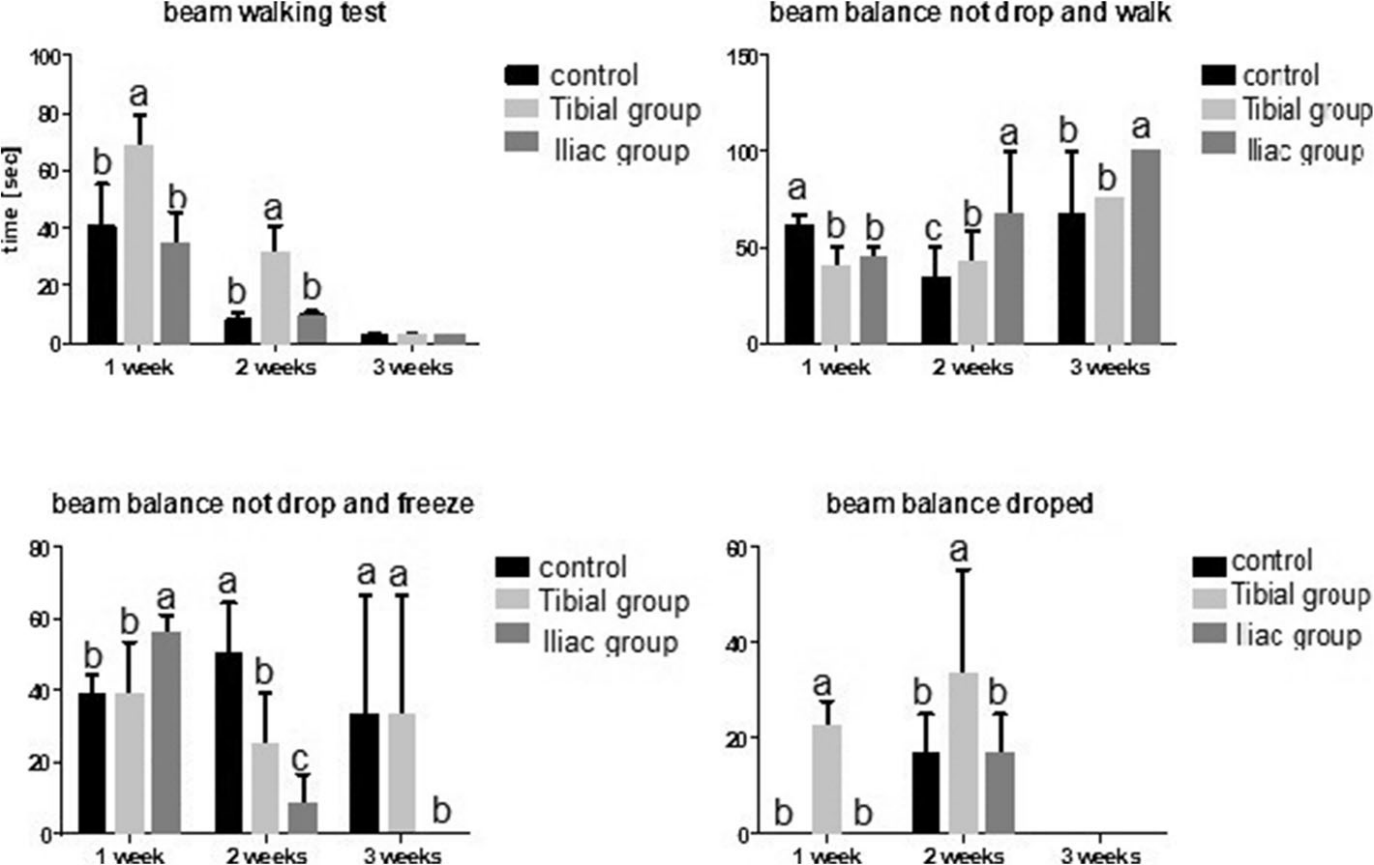
Charts summarizing the behavioral results in rats of control, tibial, and iliac groups. Rats were placed at the center of a 1.5-cm-wide square wooden bridge that was suspended 60 cm above the ground. All animal groups were tested after the injection (one trial/rat) for 3 days per week within 3 weeks and scored from 1 to 4 on beam balance. The scoring was as follows: 1, balances with freezing; 2, balances and walking; 3, hugs the beam or slips without falling; and 4, drops over the beam or hangs on the beam and falls off. The results were calculated as a percentage for each group and by ANOVA to detect significance

Rats injected intra-tibial and intra-iliac showed significant (*p*≤ 0.05) delay to traverse a narrow, elevated beam in comparison to the control rats in the first week. In the second week, enhanced recovery of rats injected intra-iliac was observed in which rats had a significant rapid traverse on the elevated beam similar to the control group. Meanwhile, there was a decreased speed of rats injected intra-tibial (Fig. 3).

#### Footprint test

Concerning stride length, it was significantly decreased (*p*≤ 0.05) in rats injected intra-tibial compared to control and rats injected intra-iliac in the first week, while in the second and third weeks post-surgery, there were no differences between the groups. Results of stride width and length revealed no significant differences in control and experimental groups during the study (Table 4).

**Table 4:** Footprint test within weeks for different groups represented by centimeters

Time	Parameter	Injection group			*P*-value
		Control	Intra-tibial	Intra-iliac	
First week	Stride length	6.64 ± 0.19^a^	5.11 ± 0.22^b^	6.25 ± 0.21^a^	0.0003
	Stride width	4.41 ± 0.13^a^	4.01 ± 0.28^a^	4.58 ± 0.2^a^	0.28
Second week	Stride length	6.16 ± 0.10^a^	6.16 ± 0.47^a^	6.41 ± 0.2^a^	0.82
	Stride width	4.8 ± 0.12^a^	4.75 ± 0.35^a^	5.5 ± 0.22^a^	0.86
Third week	Stride length	6.25 ± 0.14^a^	6.75 ± 0.38^a^	7.08 ± 0.16^a^	0.12
	Stride width	5.75 ± 0.14^a^	5.91 ± 0.36^a^	6.00 ± 0.22^a^	0.41

### Biochemical findings

**Fig. 4:**
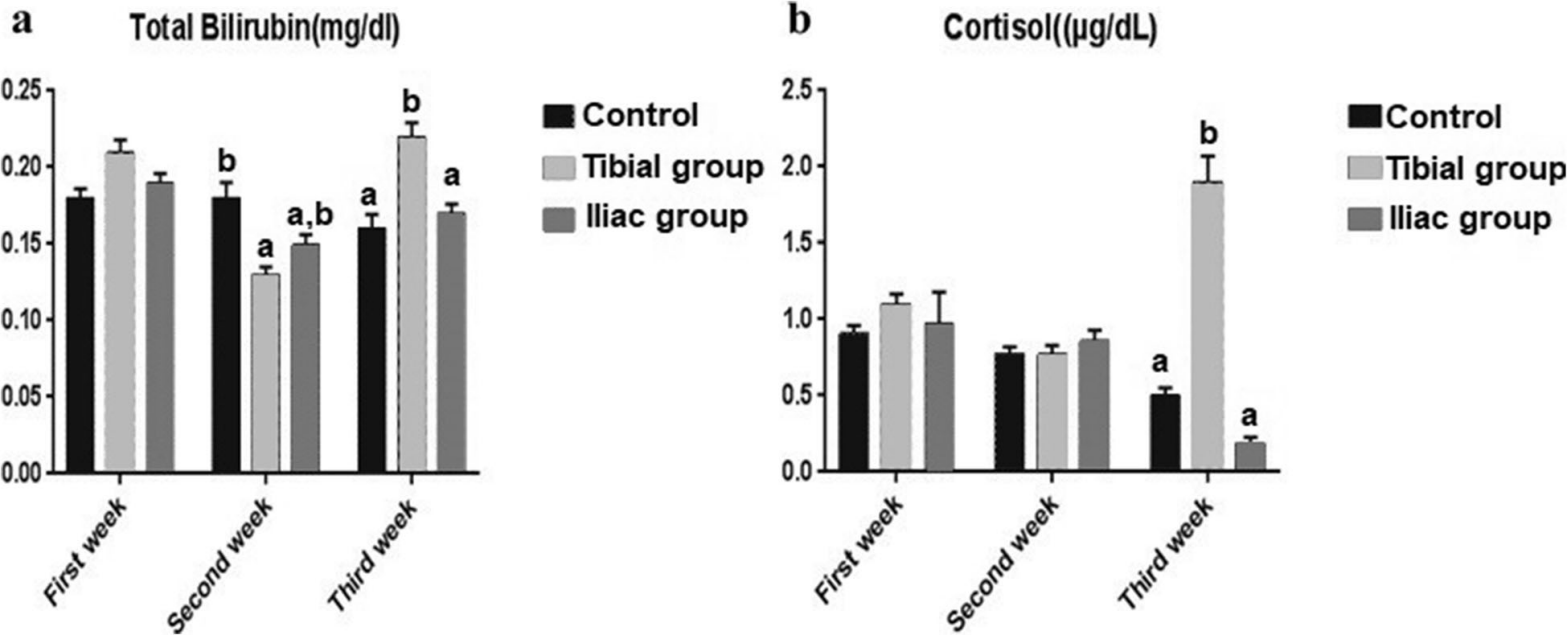
Evaluation of total bilirubin and cortisol concentrations in control, tibial, and iliac groups. **a** Total bilirubin concentration (mg/dl) and **b** cortisol concentration (μg/ dl) in control, tibial, and iliac groups. The values in the iliac group were returned to normal in a short time whereas the cortisol concentration was still high in the tibial group at the 3rd week. Results were analyzed by ANOVA test. Bars bearing different lowercase letters are significantly different at *p*≤ 0.05

The concentration of total bilirubin (mg/dL) and cortisol hormone (μg/dL) that reveal the value of stress were significantly increased up to 0.22 mg/dL and 1.9 μg/dL respectively by intra-tibial injection at third week after injection in comparison to intra-iliac injection (Fig. 4).

#### Gene expression findings

**Fig. 5:**
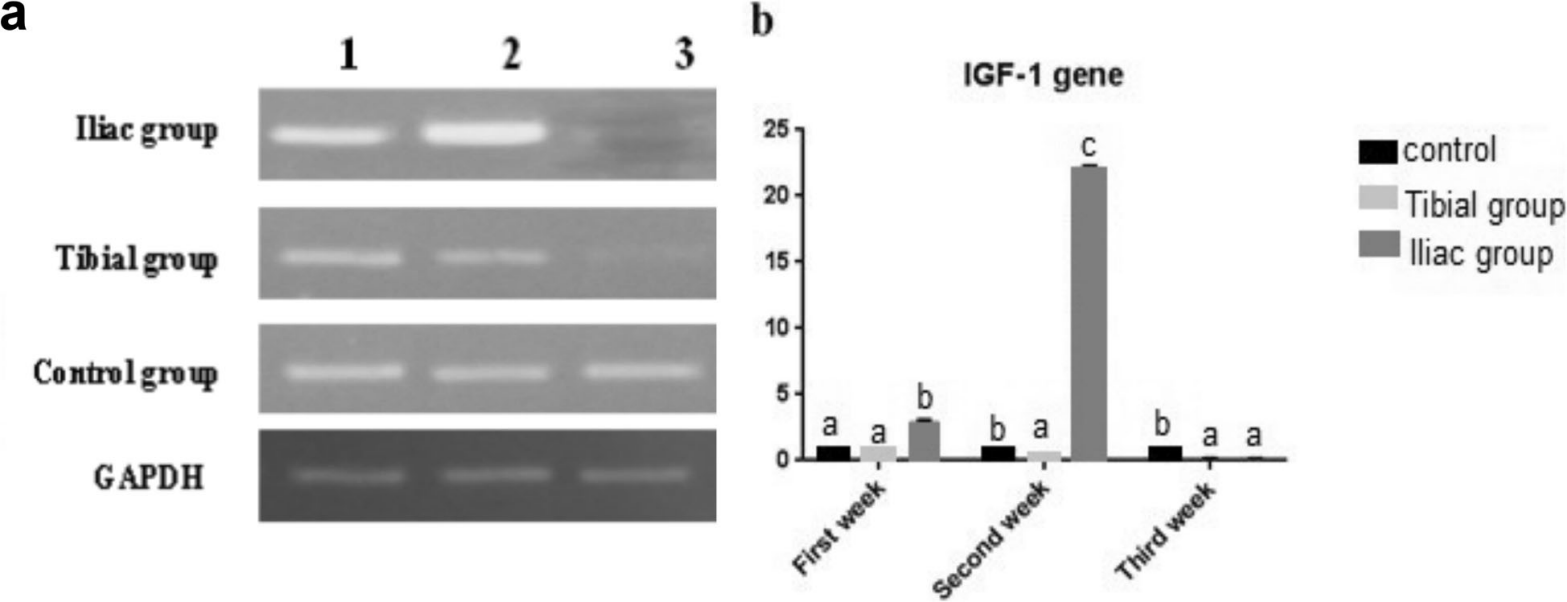
Quantitative RT-PCR of insulin-like growth factor 1 gene expression in control compared with tibial and iliac groups. **a** Electrophoretic mobility of quantitative RT-PCR products of IGF-1 and GAPDH (internal control) genes on 2% agarose gel. Lane 1, first week; lane 2, second week; and lane 3, third week. **b** Evaluation of IGF-1 gene expression in control, tibial, and iliac groups. Results were analyzed by ANOVA test. Bars bearing different lowercase letters are significantly different at *p*≤ 0.05

The expression of the IGF-1 gene in the iliac group significantly increased to 2.9, 22.1-fold in comparison to the control (*p*≤ 0.05) in the first week and second week respectively, while its expression significantly decreased in the tibial group to be 0.4-fold lower than the control group in the second week. Expression of the IGF-1 gene significantly decreased in both injection methods in the third week in comparison to the control group (Fig. 5).

### Radiological findings

Thickness estimation of the iliac crest and subchondral bone by a digimizer revealed no significant differences between the control and iliac groups all over the study period. In contrast, there were significant differences between the control group and the tibial group (Table 5).

**Table 5:** Iliac crest thickness and subchondral bone thickness of tibia

Time	Parameter		
	Ilium—ventrodorsal view	Ilium—lateral view	Tibia—ventrodorsal view
Control	0.1817±.00617	0.1713±.00467	0.0243 ± .00120^a^
First week	0.1937±.00674	0.1733±.01472	0.1257 ± .00120^c^
Second week	0.1853±.00517	0.1657±.00876	0.1340 ± .00963^c^
Third week	0.1837±.01102	0.1697±.00567	0.1002 ± .00300^b^

Letters ^a, b,^ and ^c^ within the column means significant difference at *p* ≤ 0.05 between the groups. Values are presented as mean ± SD

In the radiological findings of iliac crest, starting from day zero to different weeks of the study, the ventrodorsal view showed normal radiographic findings of the iliac crest in the control group. The ventrodorsal view of the left ilium revealed normal radiographic findings of the iliac crest at 1st week compared to the control group. In the 2nd week, the ventrodorsal view of the left ilium exhibited a sclerotic reaction of the iliac crest compared to the control group. In the 3rd week, the ventrodorsal view of the left ilium had normal radiographic findings of the iliac crest in comparison to the control group.

**Fig. 6:**
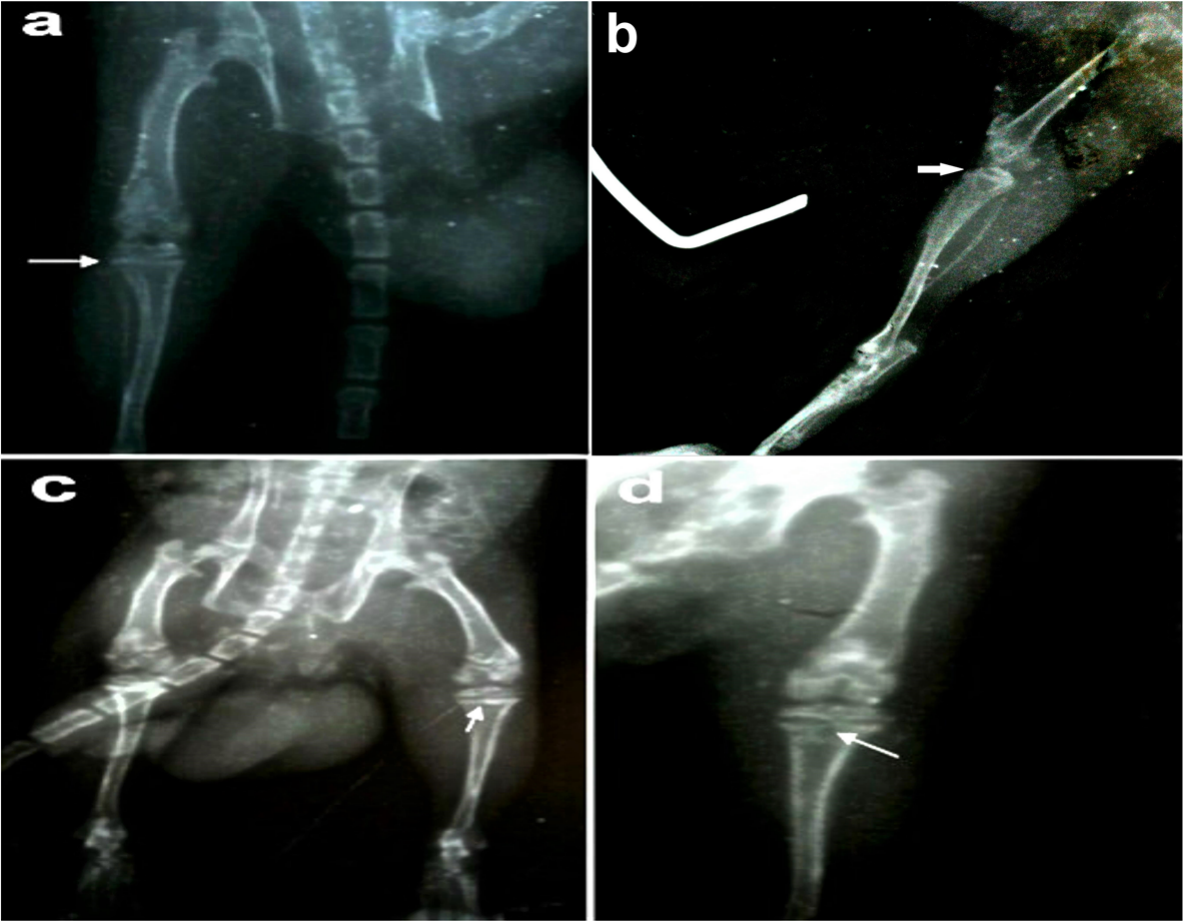
Ventrodorsal view of the left tibia at day 0 and after 1, 2, and 3 weeks showing **a** normal radiographic findings at control group, **b** subchondral reaction especially laterally at 1st, **c** mild subchondral reaction, and **d** subchondral reaction laterally and medially with subchondral bone loss at the center

Radiographic findings of the left tibia at day zero, 1st week, 2nd week, and 3rd week showed normal radiographic findings in the control group (Fig. 6a). The ventrodorsal view of the left tibia revealed a subchondral reaction especially laterally at 1st week (Fig. 6b), mild subchondral reaction at 2nd week (Fig. 6c), and subchondral reaction laterally and medially with subchondral bone loss in the center of the subchondral plate at 3rd week (Fig. 6d).

### Histopathological findings

**Fig. 7:**
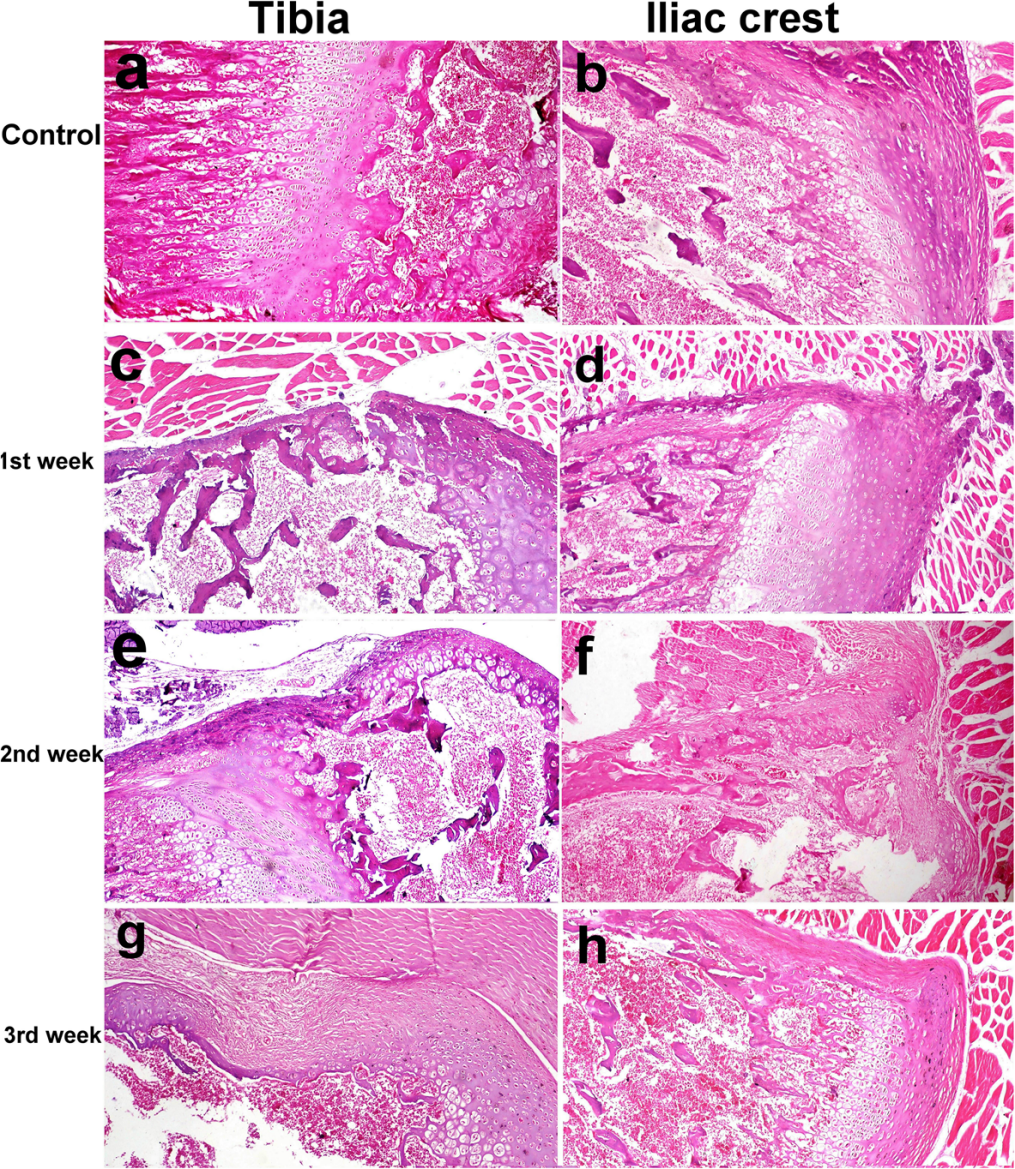
Control tibia (**a**) and iliac crest (**b**) of rats. Tibia showing discontinuity of bone, fractures of the subchondral bone, and little hemorrhage at the site of injection (**c**). Iliac crest with little bone disruption and minute fractures in the bone trabeculae at the site of injection at 1st week post-injection (**d**) (× 100). Tibia still showing fractures in the subchondral bone, degeneration, and necrosis of chondrocytes of the articular cartilage and discontinuation of the periosteum (**e**) and iliac crest showing granulation tissue formation at the site of injection at the 2nd week post-injection (**f**) (× 100). Tibia had fibrous connective tissue formation with thin bone plate formation at the site of injection (**g**). Iliac crest showed complete re-construction of bone without sign of injection (**h**) (× 100) (HE stain)

**Fig. 8:**
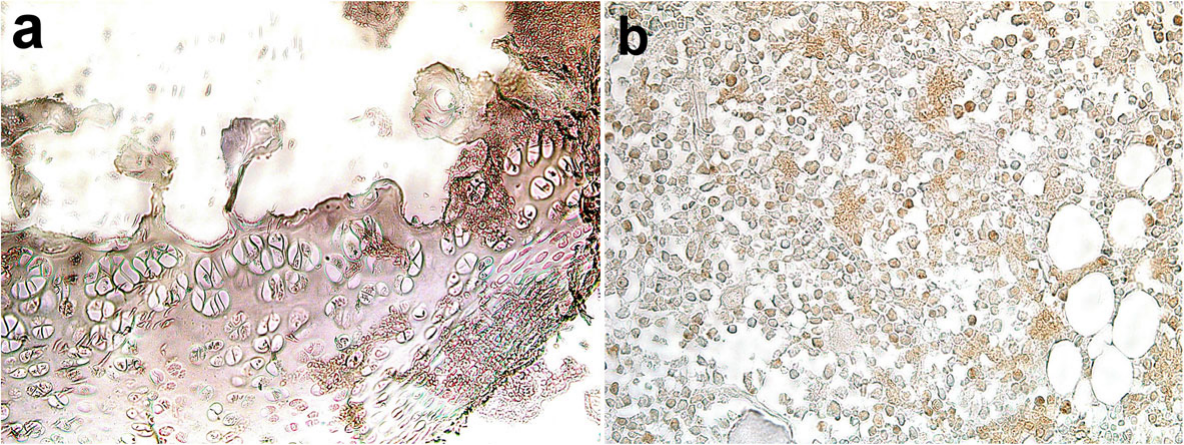
Immunohistochemistry of tibia and ilium. **a** Tibia of rat 3rd week (CD68 ×200). **b** Positive Ki67 in bone marrow cells of the ilium (Ki67 ×400)

In the control groups, the thickness and integrity of cartilage and bone in the iliac crest and tibia were normal (Fig. 7a, b). On the other hand, the injected tibia at 1 week exhibited discontinuity of bone at the site of injection with little clotted blood that was also seen in the subchondral area. Fractures of the subchondral bone were also observed (Fig. 7c). The injected iliac crest had less bone disruption than the tibia in which there were only minute fractures in the bone trabeculae with no evidence of hemorrhage (Fig. 7d). At 2 weeks post-injection, the tibia still showed fractures in the subchondral bone, degeneration, and necrosis of chondrocytes in the articular cartilage, and discontinuation of the periosteum (Fig. 7e) whereas the iliac crest began to show signs of healing in which the granulation tissue and revascularization were observed at the site of injection (Fig. 7f). At the 3rd week post-injection, the tibia showed fibrous connective tissue formation at the site of injection and a thin plate of bone underneath (Fig. 7g). In contrast, after 3 weeks, the iliac crest showed complete reconstruction of bone with little sign of previous bone injection (Fig. 7h). All semiquantitative-histopathologic lesions scores were higher in the tibia group compared to the ilium in the 3rd week (Table 6). Necrosis, disrupted trabeculae, fibrosis, and vascularization were still higher in the tibia group compared to the iliac group at the 3rd week. Moreover, via immunohistostaining, CD68-positive phagocytic cells in the tibia were found at the site of injection. Cellular proliferation was also demonstrated by Ki67 staining in the bone marrow cells of the ilium (Fig. 8).

**Table 6:** Statistical results of the semi-quantitative lesion scoring of intra-tibial and intra-iliac inoculation in all groups

Group	Tissue	Time (weeks)	Hemo.	Edema	Cong.	Infl. cell infilt.	Necrosis	Disrupted Trab.	Fibrous CT prolifer.	Capillary prolifer.	General score
**G1**	**Tibia**	**0**	**1±0**^**a**^	**1±0**^**a**^	**1±0**^**a**^	**1±0**^**a**^	**1±0**^**a**^	**1±0**^**a**^	**1±0**^**a**^	**1±0**^**a**^	**1±0**^**a**^
**G1**	**Ilium**	**0**	**1±0**^**a**^	**1±0**^**a**^	**1±0**^**a**^	**1±0**^**a**^	**1±0**^**a**^	**1±0**^**a**^	**1±0**^**a**^	**1±0**^**a**^	**1±0**^**a**^
**G2**	**Tibia**	**One**	**2.5±0.7**^**b**^	**2±0**	**3±0**^**c**^	**2.5±0.7**^**b**^	**2.5±0.7**	**3.5±0.7**^**c**^	**1±0**	**1±0**	**3.5±0.7**^**c**^
**G3**	**Ilium**	**One**	**1±0**^**a**^	**1.33±0.57**	**2±0**^**b**^	**2±0**^**b**^	**1.33±0.57**	**2±0**^**b**^	**1±0**	**1.33±0.57**	**1.66±0.57**^**b**^
**G2**	**Tibia**	**Two**	**1±0**^**a**^	**2±0**^**b**^	**3±0**^**c**^	**2±0**	**2±0**	**3±0**^**c**^	**2.5±0.7**^**b**^	**2.5±0.7**^**b**^	**3±0**^**c**^
**G3**	**Ilium**	**Two**	**1±0**^**a**^	**2±0**^**b**^	**2±0**^**b**^	**1.66±0.57**	**1.66±0.57**	**1.6±0.5**^**b**^	**2±0**^**b**^	**2.66±0.57**^**b**^	**2±0**^**b**^
**G2**	**Tibia**	**Three**	**1±0**^**a**^	**1±0**^**a**^	**1±0**^**a**^	**1±0**^**a**^	**1.33±0.57**^**a**^	**2±0**^**b**^	**2.33±0.57**^**b**^	**2.66±0.57**^**b**^	**2±0**^**a**^
**G3**	**Ilium**	**Three**	**1±0**^**a**^	**1±0**^**a**^	**1±0**^**a**^	**1.33±0.57**^**a**^	**1±0**^**a**^	**1.6±0.5** ^**a,b**^	**1.66±1.15**^**a,b**^	**1.33±0.57**^**a,b**^	**1.33±0.57**^**a**^

## Discussion

Tissue regeneration and cell replacement therapies are promising advanced treatment methods used in many diseases associated with degeneration and organ failure that remained untreated through remedies and organ replacement [29]. These include cardiovascular, neurodegenerative, and endocrine disease and chronic injuries of joints, skin, cornea, skeletal muscle, and bones [30]. Stem cells are considered one of the cell replacement therapies that have a potential role for tissue and organ repair, induce the survival of damaged tissue replacement of dying cells, and induce the generation of pluripotent stem cells [31]. Therefore, we investigated and monitored the effect of a novel technique to be used in transplantation through intra-iliac injection in comparison to intra-tibial that was used before.

In this study, low body weight gain was recorded in rats exposed to intra-tibial and intra-iliac injection in the first week post-surgery compared to the control group. An abnormal motivated locomotor behavior (lameness and abnormal gait) produced by intraosseous injection in limbs could result in a decrease in feed intake and consequently reduced body weight gains. In the second and third weeks post-surgery, the iliac group rats showed high body weight gain compared to the intra-tibial inoculated groups, which might be due to the rapid recovery and healing of intra-iliac injected rats. Open field activity monitoring assesses accurately the motor activities of rats and, therefore, is an ideal method for evaluating the degree of locomotor impairment and to assess the efficacy of elements affecting muscle function [32, 33]. The total number of squares crossed is usually used as a measure of locomotor activity, ataxia, and other gait disturbances, with high frequencies of this behavior indicating increased locomotion activities [34]. As far as we know, this is the first study to record motor behavior using the open field test to assess rats with experimentally induced intra-tibial and intra-iliac injection changes. Our results revealed that injected rats in the first week showed a decrease in peripheral activity (decrease in squares crossed) compared to the control group, as a result of postoperative suffering and pain. While in the second week, the locomotor activity of rats injected intra-iliac increased significantly (increase in squares crossed) than in the rats injected intra-tibial. Moreover, rearing at the perimeter of the enclosure has been evidenced to indicate locomotor impairment. The injected rats showed a loss in vertical activity (rearing), which mirrored their hind limb injury and severe pain and in turn inability to rear in the first week. In the second week, rats injected intra-ilium tended to be more active, displaying a higher level of vertical activity than the rats injected intra-tibial. Similarly, Rajasekaran et al. [35] demonstrated that mice exhibit reduced locomotor activity during K/BxN sera-induced arthritis. Also, Sheahan et al. [36] evaluated voluntary wheel running, locomotion, gait, social interaction, and anxiety-like behavior in two commonly used mouse models of persistent pain: Complete Freund’s Adjuvant-induced inflammation and the spared nerve injury model of neuropathic pain.

Motor performance deficits include slowing of movement, decrease in balance, and muscle strength as well as coordination difficulty [37, 38]. Tasks requiring coordinated control of motor and reflexive responses, such as the length of time an animal can traverse/balance on a wooden rod, are among tests, which attempt to assess motor incapacities and coordination skills [39]. Performance on the balance beam is a useful measure of coordination and balance in rodents. The current study revealed a poor motor coordination performance in injected rats compared to the control, which traversed the road quickly to the home cage in the first week. However, in the second week, increased motor performance and coordination were recorded in iliac group rats compared to tibial group rats. Similar results were reported previously in which pain signs such as increased lameness, decreased grip capacity, and abnormal walking patterns occurred in all groups with intra-bone marrow transplantation (IBMT) [6].

Furthermore, footprint analysis of rats was assessed to detect gait abnormalities. One week post-operation, we observed significant differences between rats injected intra-tibial and intra-iliac. The stride length of tibial group rats decreased than the rats injected intra-iliac with no change in stride width. This improvement in the gait of rats injected intra-iliac continued until the third week which might be due to the rapid recovery of iliac group rats. However, Pfeiffenberger et al. [6] indicated no differences in gait analysis (stride length and stride width) in both experimental and control groups with intra-bone marrow transplantation. The aforementioned results demonstrate that gait abnormality, inability to walk, and poor coordination are signs of pain and stress occurring postoperative in rats injected intra-tibial and intra-iliac.

Improvement in motor performance in rats injected in intra-iliac could be attributed to rapid recovery and healing as shown in histological findings in this study. Intra-bone injection in the iliac crest resulted in less bone destruction and bone loss compared to intra-tibial injection. Furthermore, the connective tissue formation was less pronounced in the iliac crest in comparison to intra-tibial injection and by three weeks, there was a complete reconstruction of bone which indicates accelerated healing. Likewise, previous studies demonstrated that tibia injection resulted in severe fibrous tissue proliferation and scar formation around the cruciate ligaments [6]. The expression of CD68 in bone tissue was reported to be associated with bone marrow macrophages and osteoclasts and is believed to be crucial to normal skeletal physiology [40]. Osteal macrophages support bone formation and remodeling and mediate parathyroid hormone-dependent bone regeneration [41]. In the current study, CD68+ macrophages were observed in the tibia suggesting excessive damage to the bone which in turn required the presence of macrophages at the wound site. Ki-67-positive cells were observed only in bone marrow cells in the iliac group rats which are considered a normal finding. These results are similar to a previous study which showed that Ki-67-positive cells were rarely noticed in cancellous fracture and normal bone tissue suggesting that the cancellous fracture repair might not take place by local cells, such as mesenchymal stem cell (MSC) [42]. Another study showed that there was an immediate increase in MSC relative levels following skin/muscle laceration and femoral bone defect injuries which might be followed by migration to the sites of injury as tissue repair progresses [43].

Acute postoperative pain can lead to a wide range of adverse effects, such as anxiety, depression, restlessness, and sleep deprivation [44]. Behavioral activities include anxiety, measurement in the open field test, freezing time, and the number of fecal boli [45]. All parameters were greatly influenced by intraosseous injection in rats. Significantly increased time of freezing and immobility in the injected rats in the open field in the first week post-surgery is characteristic of an increased level of anxiety, fear, and pain which was improved in the iliac group rats by the 2nd and 3rd weeks as a sign of rapid recovery. In contrast, tibial group rats showed an increase in the freezing time with slow recovery. Increased immobility in the open field is characteristic of an increased level of anxiety [46]. Fecal boli (defecation) increase was shown to be a sensitive measure for the anxiety state of animals. In this study, a high level of anxiety was observed in injected rats in the first week as a result of pain. In the second week, rats injected via the intra-iliac route became less anxious due to the relief of pain rapidly. Taken together, these reports and the results presented here, it can be concluded that intra-osseous injection in rats produces anxiety-like behavior and fear because of suffering and pain [47, 48]. Therefore, we recommend using operative analgesia for pain relief and reducing discomfort and stress.

To monitor the suffering and pain of the animals after intra-tibial and iliac injection, we assessed the concentration of total bilirubin and stress hormone in serum. Bilirubin is the end product of biliverdin oxidation due to hemoproteins degradation under the effect of stress inducible enzyme; heme oxygenase-1 [49]. In the present study, the concentration of total bilirubin and cortisol significantly increased in the serum of intra-tibial groups compared to intra-iliac one, that agreed with Pfeiffenberger et al. [6] who reported an intra-osseous transplantation through tibial shaft is technically easier, but the degree of impairment and distress were consistently higher than intra-femoral transplantation.

Insulin-like growth factor (IGF) is a peptide hormone that binds to a specific IGF binding protein (IGFBP-3). IGFBP-3 intensifies the effect of IGF-1 by prolonging its half-life time and promoting its trans-endothelial transportation to target tissues [50]. IGF-1 circulatory level is regulated by growth hormone which induces its hepatic expression (70–90% of circulatory IGF-1) and its extra-hepatic tissue expressions such as in bone, brain, lung, muscle, uterus, ovaries, adipose tissue, and bone marrow stromal cells in a paracrine/autocrine fashion [51, 52]. IGF-1 is one of the essential and fundamental hormone concomitants with growth hormones for cellular growth, differentiation, survival, cell cycle propagation, and skeletal maturation during the postnatal period to adult life for bone maintenance [13, 53].

In the present study, we quantified the expression of the IGF-1 gene in the three groups, which indicated a significant elevation of its expression in the intra-iliac injection group compared to others. Similarly, Mohan and Kesavan [54] monitored the impact of IGF-1 in bone strength through regulation of its size and mineral density under the effect of systemic hormones, local growth factors, and mechanical stress. IGF-I and II are synthesized and retained in the bone to stimulate DNA, collagen, and non-collagenous protein [55]. Different studies hypothesized the effects of IGF-1 in bone metabolism through its resorption and formation, while its lower content results in bone fracture [12, 56]. Besides IGF-1 growth-promoting action, it induces the regeneration of wound epithelial cells and ameliorates endothelial damage by inhibiting its activation [57, 58]. IGFs play an important role in stem cell biology by promoting self-renewal, proliferation, and differentiation [14]**.** A high level of IGF-1 has anti-inflammatory properties by balancing the ratio between pro-inflammatory/anti-inflammatory cytokines through reducing the pro-inflammatory site [59]. Accordingly, we hypothesized that the elevation of IGF-1 gene expression in iliac group rats has a protective role in attenuating injection damages and promoting the regeneration of cells at the site of injection.

In conclusion, the adopted refinement of intra-iliac injection elevated the IGF-1 gene expression in rats which accelerates healing and had a low impact on the animal behavior, radiological finding, and histopathology compared to tibial injection. Consequently, the intra-iliac injection could be a promising technique for intra-bone injection in rats used in preclinical experiments of hematopoietic or stem cell transplantation or bone marrow aspiration.

## Data Availability

The datasets used and/or analyzed during the current study are available from the corresponding author on reasonable request.
